# Enhanced Visible Transmittance of Thermochromic VO_2_ Thin Films by SiO_2_ Passivation Layer and Their Optical Characterization

**DOI:** 10.3390/ma9070556

**Published:** 2016-07-09

**Authors:** Jung-Hoon Yu, Sang-Hun Nam, Ji Won Lee, Jin-Hyo Boo

**Affiliations:** 1 Department of Chemistry, Sungkyunkwan University, Suwon 440-746, Korea; thank42@hanmail.net (J.-H.Y.); ljw9917@naver.com (J.W.L.); 2 Institute of Basic Science, Sungkyunkwan University, Suwon 440-746, Korea; askaever@gmail.com

**Keywords:** VO_2_, thermochromic, SiO_2_ passivation

## Abstract

This paper presents the preparation of high-quality vanadium dioxide (VO_2_) thermochromic thin films with enhanced visible transmittance (T_vis_) via radio frequency (RF) sputtering and plasma enhanced chemical vapor deposition (PECVD). VO_2_ thin films with high T_vis_ and excellent optical switching efficiency (E_os_) were successfully prepared by employing SiO_2_ as a passivation layer. After SiO_2_ deposition, the roughness of the films was decreased 2-fold and a denser structure was formed. These morphological changes corresponded to the results of optical characterization including the haze, reflectance and absorption spectra. In spite of SiO_2_ coating, the phase transition temperature (T_c_) of the prepared films was not affected. Compared with pristine VO_2_, the total layer thickness after SiO_2_ coating was 160 nm, which is an increase of 80 nm. Despite the thickness change, the VO_2_ thin films showed a higher T_vis_ value (λ 650 nm, 58%) compared with the pristine samples (λ 650 nm, 43%). This enhancement of T_vis_ while maintaining high E_os_ is meaningful for VO_2_-based smart window applications.

## 1. Introduction

For improved design and practicality, the exterior of many buildings has been changed into full-window constructions in recent years [1]. However, such window architecture systems lead to more cooling and heating energy loss in the summer and winter seasons. One of the best ways to solve this problem is vanadium dioxide (VO_2_) based thermochromic smart windows. VO_2_ is a well-known material that undergoes a fully reversible metal-insulator phase transition (MIT) at 68 °C accompanied by structural changes from a monoclinic (semiconductor with high IR transparency) to a rutile (metallic form with IR reflection) phase [2,3]. These unique properties make VO_2_ a promising material for application in energy saving smart windows with solar heat control [4,5,6,7,8]. However, for practical applications, several challenges must be improved: the transition temperature (T_c_) is too high to apply on real field; the weak optical contrast in the IR region; the luminous transmittance (T_lum_) is less than ~40% for films; the unfavorable color (yellow/brown). Up to now, there have been many efforts to improve the above issues [9,10]. More than anything, low visible transmittance (T_vis_) is the most critical drawback of VO_2_ for applications in glazing systems. The low T_vis_ originates from strong innerband and interband absorption in the short-wavelength range for both the metallic and semiconductive states [11,12]. To enhance the T_vis_, band gap adjustment by doping has been attempted. Zhou et al. reported on the preparation of Mg-doped VO_2_ nanoparticles via hydrothermal synthesis [13]. By controlling the Mg doping contents, they demonstrated the absorption edge of VO_2_ particles with a blue-shift from 490 to 440 nm at a Mg content of 3.8 at %, representing a widened optical band gap from 2.0 eV for pure VO_2_ to 2.4 eV with 3.8 at % doping. Similar research was reported by Chen et al., who fabricated Ti-doped VO_2_ nanoparticles with successful improvement of T_vis_ by up to 53% via a hydrothermal process [14]. Zhou et al. reported another approach to improve the T_vis_ of VO_2_ thin films by designing VO_2_ thin films with a periodic porous structure [15]. These periodic porous thermochromic VO_2_ thin films were fabricated via a colloidal lithography approach and exhibited a high T_vis_ (81% maximum). However, in spite of the significant enhancement of the T_vis_, the optical switching efficiency (E_os_) of previously reported VO_2_ thin films is still lower that of traditional low-emission glass. Moreover, it is difficult to control the stoichiometry of a vanadium-oxygen system using the colloidal lithography approach because organic residues from the colloid might influence the valence of VO_2_ as well as the phase transformation during the annealing process. VO_2_-based multi-layered structures such as VO_2_/ZrO_2_ double layers [16] and TiO_2_/VO_2_/TiO_2_ triple layers [17] are the most effective solutions to the above problems. These antireflective layers including ZrO_2_ and TiO_2_ can protect VO_2_ from oxidation and provide new functions such as photocatalysis in addition to improving the visible transmittance. However, to date, the reported results on applying an anti-reflective layer have been based on more complex procedures or solution processes for which additional annealing is essential. Thus, the reported VO_2_ thin films contain low E_os_ originating from inconsistent stoichiometry due to additional annealing or other procedures [18,19].

In this work, high-quality VO_2_ thermochromic thin films with significantly enhanced T_vis_ were prepared by applying a SiO_2_ layer using plasma enhanced chemical vapor deposition (PECVD). This SiO_2_ layer deposited by the PECVD method has advantages such as the lack of additional post-annealing processes. Thus, damages from oxidation or reduction, which can influence the crystallinity of VO_2_, do not occur during SiO_2_ deposition. In addition, high uniformity and reproducibility underscore the attractiveness of PECVD. This paper also explains how the improvement of T_vis_ was identified through optical analysis.

## 2. Materials and Methods

### 2.1. SiO_2_/VO_2_ Thin Film Preparation

VO_2_ thin films were prepared by RF magnetron sputtering with VO_2_ ceramic targets (Taewon Scientific, Seoul, Korea, 99.9%, 2 inch). Before deposition, the Eagle XG glass (Corning, New York, NY, USA, 2.5 × 2.5 cm^2^) used as a substrate was cleaned ultrasonically with hydrochloric acid (1 M) and Ethanol, then was subsequently dried with N_2_. The vacuum chamber was evacuated to 1.2 × 10^−5^ Torr, and Ar gas was introduced with 150 sccm. The RF power, working pressure, and distance of the target to the substrate were maintained at 180 W, 46 mTorr and 40 mm, respectively. The deposition time for all samples was fixed at 15 min. The prepared VO_x_ thin films were crystallized via post-annealing under vacuum conditions (10 mTorr) at a temperature of 575 °C for 4 h with a heating rate of 30 °C/min. The SiO_2_ layer was deposited by PECVD onto the as prepared VO_2_ thin films under a working pressure of 200 mTorr. Hexamethyldisilazane (HMDSN) was used as a precursor and a thickness of 80 nm was achieved for the SiO_2_. More detail procedure and conditions of PECVD is described elsewhere [20].

### 2.2. Characterizations

The surface morphologies of the films were determined using FE-SEM (JEOL, JSM-7100 F, Tokyo, Japan) and AFM (PARK system, XE-100, Suwon, Korea). Crystallization information for the films was determined using X-ray diffraction (XRD) (Bruker D8 Advance system, Billerica, MA, USA) with Cu Kα radiation (λ = 1.5416 Å). Diffraction patterns were collected for 2θ values between 10° and 80° with a 2° glancing angle, and scanned at a rate of 5°/min. The phases present were identified by comparing the peak intensities and their corresponding 2θ values to various vanadium oxide standards using the software PCPDFWIN ver. 2.1 (JCPDS-ICDD, Philadelphia, PA, USA). Raman spectra were collected with a confocal Raman microscope (Witec, ALPHA 300 M, Ulm, Germany) based on the 532 nm CO_2_ laser. The optical and thermochromic properties of the films were measured at the temperature range between 20 to 100 °C by using a UV-vis-NIR spectrometer (SHIMADZU, UV-3600, Kyoto, Japan) equipped with handmade heating units including a PID temperature controller. For all samples, the integral visible transmittance (T_lum_, 390–830 nm) and solar transmittance (T_sol_, 280–2500 nm) was obtained based on the earlier publication [21].

## 3. Results and Discussion

**Figure 1:**
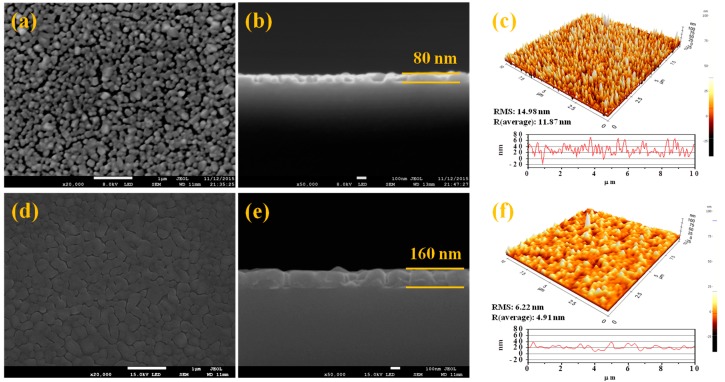
SEM and AFM images of VO_2_ (**a**–**c**) and SiO_2_/VO_2_ (**d**–**f**) thin films. The average roughness of the samples was (**c**): 11.87 nm and (**f**): 4.91 nm.

Figure 1 shows the SEM and AFM images of surface morphologies for the pristine VO_2_ and SiO_2_/VO_2_ thin films. For the pristine VO_2_, the mean grain size and film thickness were around 150 nm and 80 nm, respectively. Moreover, the clearly formed grain boundary was attributed to the surface roughness value of 11.87 nm. On the other hand, changes in the surface morphology of the films with denser and smoother grain boundaries, including those with 160 nm of total thickness, were observed after SiO_2_ coating. In addition, an average roughness value of 4.91 nm was obtained, which is a 2-fold decrease compared to pristine VO_2_. The obtained results suggest that the empty space between the grain boundary of pristine VO_2_ was filled with SiO_2_ during the PECVD process.

**Figure 2:**
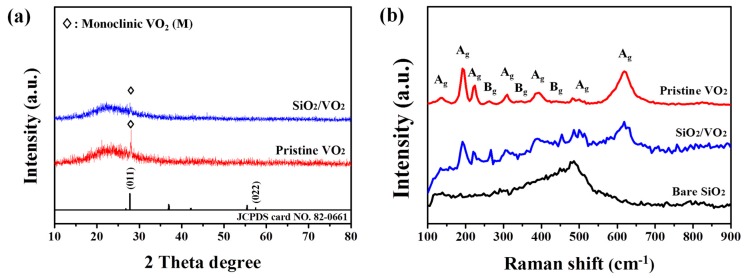
X-ray diffraction (XRD) (**a**) and Raman spectra (**b**) of VO_2_ and SiO_2_/VO_2_ thin films.

**Figure 3:**
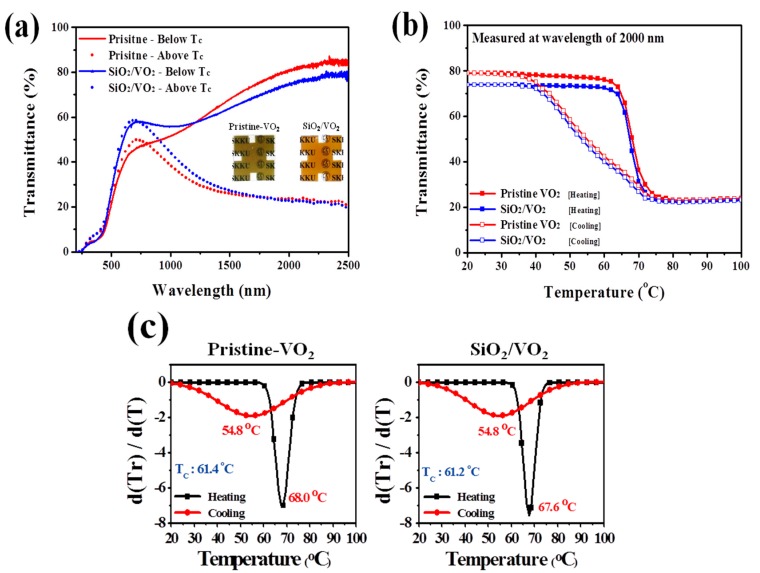
Transmittance spectra (**a**); hysteresis loops at 2000 nm (**b**) as well as corresponding d(Tr)/d(T)&T curve (**c**) for pristine VO_2_ and SiO_2_/VO_2_ thin films. The inset images in (**a**) correspond respectively to photographs of the pristine VO_2_ film (left) and VO_2_/SiO_2_ film (right). In (**a**), solid line measured at 25 °C, dashed line at 100 °C.

Figure 2 shows the XRD data and Raman spectra for each sample. The XRD pattern showed weak signal intensities for the crystallite, resulting from the short-range ordered nanocrystallinity and thinness of the films. The broad shoulder within 15°–40° is due to the contribution of the amorphous SiO_2_ substrate [22]. A peak at 27.8° that can be ascribed to the (011) plane of monoclinic VO_2_ (JCPDS no. 82-0661) was observed in the pristine VO_2_ thin films. For the SiO_2_/VO_2_ thin films however, decreased peak intensity was observed, originating from disruption of the thickly covered SiO_2_ film. Most importantly, no clear diffraction peaks for other vanadium oxides were observed. Raman models corresponding to VO_2_ (M) appeared in each sample with peaks centered at 135, 192, 225, 263, 308, 338, 392, 440, 499, and 617 cm^−1^ [23]. The change in Raman shifts for the films was within the measurement accuracy (±2 cm^−1^). For pristine VO_2_ thin film, peaks from other types of vanadium oxide were not observed. Moreover, the peaks around 195 cm^−1^ and 225 cm^−1^ corresponded to A_g_ symmetry vibrational modes, which disappeared in VO_2_ (R) [24,25]. These two vibrational modes play a decisive role in the structural transition of VO_2_. Thus, the appearance of strong peaks suggests high optical switching characteristics. After SiO_2_ coating, however, a broad peak around 480 cm^−1^ attributed to amorphous SiO_2_ was observed with a moderate signal to noise ratio while the initial peaks corresponding to VO_2_ (M) remained. In other words, SiO_2_ coating via PECVD does not affect the crystallinity of VO_2_ thin films. The corresponding evidence is shown in Figure 3b,c. The hysteresis loop at 2000 nm was obtained from the optical transmittance of each sample as a function of temperature, and a plot of d(Tr)/d(T)&T was obtained from one peak with a well-defined maximum. Each of the d(Tr)/d(T)&T curves were analyzed with a Gaussian function using the single peak fitting module in Origin pro 8.0 software (Originlab, Washington, DC, USA). Distinguishable changes in T_c_ and hysteresis width, which are closely related to the crystallographic orientation [26], were not observed except for a slight decrease in transmittance.

**Figure 4:**
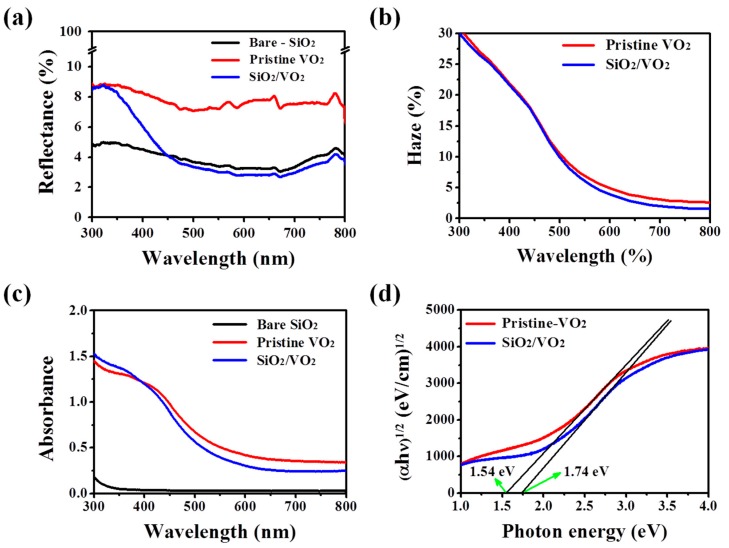
Optical analysis data for each film based on the reflectance (**a**); haze (**b**); absorbance (**c**) and optical band gap graph (**d**).

The optical analysis results for each sample are shown in Figure 3a. Transmittance results demonstrate the thermochromic properties of each sample measured at 20 °C (solid line) and 100 °C (dashed line). The E_os_ of the SiO_2_/VO_2_ films was 51.7%, which is slightly lower compared with pristine VO_2_ thin films (57.1%), but the difference is not significant. The SiO_2_/VO_2_ thin films showed a significantly enhanced T_vis_ value (λ_650 nm_, 58%) compared with the pristine samples (λ_650 nm_, 43%). In addition, the inset images in Figure 3a clearly show a contrast change after SiO_2_ coating. Though the SiO_2_/VO_2_ film is thicker, it exhibited higher T_vis_ than the pristine VO_2_. This enhancement of T_vis_ can be attributed to two factors. Furthermore, these optical results can be defined by average *T*_lum_((*T*_lum, 20 °C_ + *T*_lum, 100 °C_)/2) and ∆*T*_sol_(*T*_sol, 20 °C_ − *T*_sol, 100 °C_). The average *T*_lum_ of pristine and SiO_2_/VO_2_ films are 37.6% and 47.7%, respectively, and ∆*T*_sol_ are 8.06 and 7.62. One is the difference in reflectance between pristine VO_2_ and SiO_2_/VO_2_ thin films in the visible region as shown in Figure 4a. As previously mentioned, the formation of smoother and denser grain boundaries after SiO_2_ coating could reduce the surface roughness, which can influence the decrease of light scattering on the surface observed in SEM images. Therefore, the reflectance for the SiO_2_/VO_2_ thin films was decreased from 8% to 4% compared to the pristine VO_2_ thin films. However, the change in the reflectance is not enough to confirm the enhancement of T_vis_ for SiO_2_/VO_2_ thin films. Figure 4b shows the haze for each sample in the visible region. For the SiO_2_/VO_2_ thin films, a slight decrease in haze over 500 nm was observed but other considerable changes did not appear. For this reason, more evidence is needed to validate the transmittance results.

Figure 4c shows the absorbance data for each sample measured in the visible region. The data obviously confirm the change in the absorption edge toward the blue region and a decrease in absorbance after SiO_2_ coating. The relative optical band gap for each sample is shown in Figure 4d, and the results correspond to the results for reflectance and absorbance. The absorption coefficient α was estimated using the transmittance data for the two films [27].

α = (1/∆d)ln(T_1_/T_2_) (1)

where ∆d is the thickness of pristine VO_2_ or SiO_2_/VO_2_, T_1_ is the transmittance of the substrate (Eagle glass) and T_2_ is the transmittance of each sample. The optical band gap was determined with the following formula [28].

(αhν)^1/2^ ∝ (E − E_g_) (2)

linear extrapolation of (αhν)^1/2^ vs. hν near the band gap provided E_g_ as the intercept at the α = 0 axis. The optical band gap for the pristine VO_2_ thin film increased from 1.54 to 1.74 eV after SiO_2_ coating. This widening of the optical band gap induces a blue shift and an enhanced visible transmittance [15]. This means that the enhancement of T_vis_ for SiO_2_/VO_2_ corresponds to the decrease in absorbance and reflectance including the formation of denser and smoother surfaces on the films.

## 4. Conclusions

In this paper, high-quality VO_2_ thermochromic thin films with enhanced T_vis_ up to 58% were successfully prepared with the addition of a SiO_2_ layer. The results indicate that the enhanced T_vis_ was due to a reduction of surface roughness with a blue shift in the absorption spectra observed after SiO_2_ coating. In addition, the SiO_2_/VO_2_ thin films had high E_os_ and the crystallographic orientation was maintained. This enhancement of T_vis_ while maintaining high E_os_ is meaningful for VO_2_-based smart window applications.

## Abbreviations

The following abbreviations are used in this manuscript:

PECVD	Plasma enhanced chemical vapor deposition
T_vis_	Visible transmittance
E_os_	Optical switching efficiency
VO_2_	Vanadium dioxide
MIT	Metal-insulator transition
SEM	Scanning electron microscopy
XRD	X-ray diffraction
